# The AmAZI1ng Roles of Centriolar Satellites during Development

**DOI:** 10.1371/journal.pgen.1004070

**Published:** 2013-12-26

**Authors:** Moe R. Mahjoub, Meng-Fu Bryan Tsou

**Affiliations:** 1 Renal Division, Department of Medicine, Washington University, St. Louis, Missouri, United States of America; 2 Department of Cell Biology and Physiology, Washington University, St. Louis, Missouri, United States of America; 3 Cell Biology Program, Memorial Sloan-Kettering Cancer Center, New York, New York, United States of America; Washington University School of Medicine, United States of America

**Figure 1:**
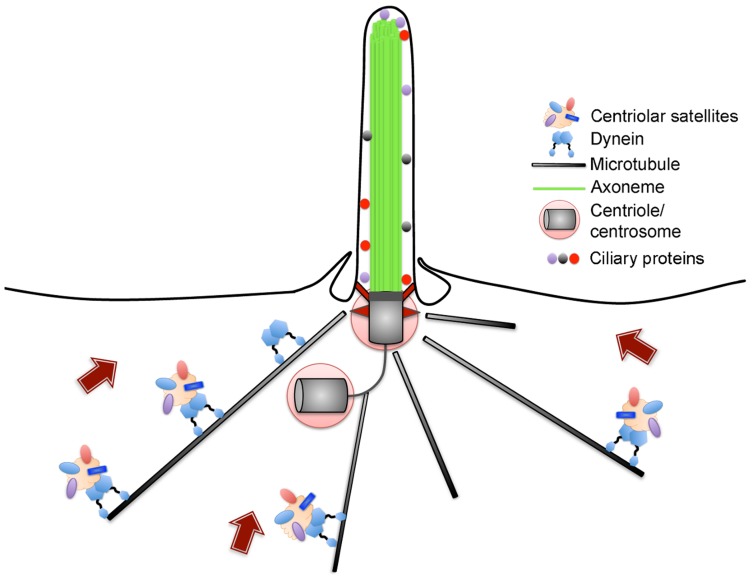
Schematic of centriolar satellites undergoing microtubule-dependent movement towards the centrosome and cilium. Satellites are composed of a number of proteins, including Azi1, which are typically found in a region surrounding the centrosome. Satellites mediate the trafficking of some proteins destined for the cilium but can also inhibit the movement of other proteins to the centrosome-cilium complex.

The precise trafficking and spatial organization of signaling molecules within cells is critical for many fundamental cellular processes. Two interconnected microtubule-based organelles, the centrosome and primary cilium, have been making headlines recently due to their role as central “hubs” for coordinating such signaling events. The centrosome is the major microtubule-nucleating center in animal cells, which polarizes microtubule arrays and thereby directs microtubule-based trafficking toward itself and its associated structure, the primary cilium. [1]. The primary cilium is a tiny hair-like sensory organelle that is templated by one of two centrioles, core elements of the centrosome, and protrudes above the apical surface of almost every cell in the human body (Figure 1). Together, the centrosome and cilium mediate the initiation and transmission of extracellular signals to the interior of the cell, thus controlling many aspects of cell physiology [2], [3]. Defects in the structure and/or function of these organelles result in human disease conditions termed “ciliopathies,” a heterogeneous group of disorders with phenotypes including cystic kidneys; digit, bone, and brain anomalies; infertility; and even cancer [4], [5].

Human diseases associated with centrosome-cilium aberrations generally result from defects in the proper assembly of the centrosome or cilium, or the trafficking of proteins to these structures. It is now well established that movement of proteins to and from the centrosome-cilium complex is facilitated by centriolar satellites, non-membranous 70–100 nm cytoplasmic granules that concentrate around the centrosome in animal cells (Figure 1) [6]. Centriolar satellites are generally defined by PCM1, a large scaffolding protein reported to function in dynein-dependent, microtubule-based trafficking of centrosomal and ciliary proteins [7], [8]. Although the proteome of centriolar satellites remains unknown, a number of proteins have now been shown to associate with PCM1, many of which are mutated in patients with ciliopathies [9]–[14]. However, the functional significance of centriolar satellites in mammalian development and ciliogenesis in vivo remains unclear. In this issue, Hall and colleagues [15] address the role and requirement of Azi1, a conserved satellite protein, in regulating cilium function during development.

Azi1 was originally identified as a centrosomal protein [16] and localized to centriolar satellites [17]. Hall et al. spatially refined the localization pattern of mouse Azi1, and discovered that Azi1 is fairly dynamic and undergoes microtubule-based trafficking to and from the centrosome. They noted higher levels of Azi1 around centrioles and surrounding centriolar satellites of ciliated cells compared to non-ciliated cells, and identified a further pool of Azi1 at the transition zone, an area at the base of cilia involved in regulating protein entry into the ciliary space [18]. Together, these results show a redistribution of Azi1 upon initiation of ciliogenesis, hinting at a functional role in regulating ciliary protein trafficking. Indeed, Azi1 was previously shown to be essential for ciliogenesis in zebrafish and flies [19], [20], and a siRNA screen in human cells also identified Azi1 as necessary for cilium formation [21]. Similarly, Hall et al. find that knockdown of Azi1 in mouse cells using siRNA, which they refer to as “acute” loss of the protein, results in a significant proportion of cells that lack primary cilia. Given the high conservation of Azi1 among ciliated organisms, the loss-of-function phenotypes from various model organisms, as well as the siRNA results, it was safe to predict that Azi1 would have a central role in mammalian cilia biology in vivo. Thus, the authors generated mouse mutants null for Azi1 to test the role of this protein (and centriolar satellites) in mammalian development.

Remarkably, Azi1 null mice are viable, and display none of the gross abnormalities commonly associated with either primary or motile cilia dysfunction. Examination of primary mouse embryonic fibroblasts (MEFs) isolated from Azi1 null mice showed that formation and compartmentalization of primary cilia appear normal, with normal distribution of ciliary membrane and transition zone proteins. Similarly, there were no gross defects with regard to centrosome duplication or centriolar satellite composition and distribution in cells lacking Azi1. Ultrastructure analysis of motile multiciliated tracheal epithelia also failed to detect any defects in centrioles/basal bodies and cilia. Collectively, and in stark contrast with the acute Azi1 loss in human and mouse cells, primary and motile cilia structure and function appear grossly normal in Azi1 null animals in vivo, as well as in Azi1 null MEFs grown in vitro.

The difference in phenotypes observed following acute knockdown of Azi1 versus permanent deletion of the gene (referred to as “chronic” loss by the authors) is suggestive of a compensation mechanism that, in essence, rescues the defects caused by loss of Azi1. Intriguingly, while such compensation was seen in most tissues, Azi1 null male mice were infertile, exhibiting post-meiotic defects in spermatogenesis. Sperm cells lacked flagella and any remaining axonemes were truncated and immotile, likely due to mistrafficking of proteins required for flagellar assembly. This indicates that Azi1 (and by extension centriolar satellites) plays a critical role in regulating trafficking of proteins in the highly specialized sperm cells that, unlike other ciliated cell types, cannot compensate for loss of Azi1. This tissue-specific phenotype is reminiscent of human mutations in a number of genes required for centrosome assembly and duplication, where the predominant phenotype appears to be microcephaly [22]–[26]. These latter findings suggest that, during development, most tissues can compensate and overcome mutations in centrosomal proteins, except for neural progenitor cells, which appear most sensitive to these perturbations. The findings by Hall et al. similarly indicate that sperm cells may be especially sensitive to mutations in centriolar satellite genes.

The functional compensation reported by Hall et al. highlights the complexity and challenge in understanding cilia assembly and function in the context of a developing organism. The basis of the compensation, at this point, is unclear, as it cannot be simply attributed to genetic redundancy, but a mechanistic understanding of the process will be critical if more cases like Azi1 are discovered in the future. Interestingly, the Azi1 null MEFs isolated by Hall et al. are shown to be “active” in compensating for the loss of Azi1; it will be very useful to test whether the compensation can be reversed (or switched on/off) by inducibly expressing exogenous Azi1 in these cells. One interesting strategy would be to utilize the Azi1 null MEFs as an effective in vitro system to understand how functional compensation works in this case, or with centriolar satellites in general. Moreover, this study also highlights the importance of functional follow-up studies of siRNA-based data and cautions against direct extrapolation of transient loss-of-function phenotypes to the genetic in vivo phenotype. On the other hand, transient loss-of-function studies may expose roles of genes that would otherwise be overlooked due to compensation in long-term mutagenesis studies in vivo. As such, it will be important to combine both approaches in future studies.
